# Influential factors and transcriptome analyses of immature diploid embryo anthocyanin accumulation in maize

**DOI:** 10.1186/s12870-022-03971-5

**Published:** 2022-12-24

**Authors:** Chen Chen, Yuling Zhang, Xiuyi Fu, Chuanyong Chen, Shanshan Wu, Chunyuan Zhang, Huasheng Zhang, Yiyao Chang, Shaojiang Chen, Jiuran Zhao, Chenxu Liu, Yuandong Wang

**Affiliations:** 1 Maize Research Institute, Beijing Academy of Agriculture and Forestry Sciences, Beijing, 100097 China; 2 National Maize Improvement Center of China, College of Agronomy and Biotechnology, Sanya Research Institute, China Agricultural University, Beijing, 100193 China

**Keywords:** Anthocyanin biosynthesis, Haploid identification, Immature haploid embryos, Maize

## Abstract

**Background:**

Anthocyanins are widely applied as a marker for haploid identification after haploid induction in maize. However, the factors affecting anthocyanin biosynthesis in immature embryos and the genes regulating this process remain unclear.

**Results:**

In this study, we analyzed the influence of genetic background of the male and female parents, embryo age and light exposure on anthocyanin accumulation in embryos. The results showed that light exposure was the most crucial factor enhancing the pigmentation of immature embryos. The identification accuracy of haploid embryos reached 96.4% after light exposure, but was only 11.0% following dark treatment. The total anthocyanin content was 7-fold higher in immature embryos cultured for 24 h under light conditions compared to embryos cultured in the dark. Transcriptome analysis revealed that the differentially expressed genes between immature embryos cultured for 24 h in dark and light chambers were significantly enriched in the pathways of flavonoid, flavone, flavonol and anthocyanin biosynthesis. Among the genes involved in anthocyanin biosynthesis, five up-regulated genes were identified: *F3H*, *DFR*, *ANS*, *F3′H* and the MYB transcription factor-encoding gene *C1*. The expression patterns of 14 selected genes were confirmed using quantitative reverse transcription-polymerase chain reaction.

**Conclusion:**

Light is the most important factor facilitating anthocyanin accumulation in immature embryos. After 24 h of exposure to light, the expression levels of the structural genes *F3H*, *DFR*, *ANS*, *F3′H* and transcription factor gene *C1* were significantly up-regulated. This study provides new insight into the factors and key genes regulating anthocyanin biosynthesis in immature embryos, and supports improved efficiency of immature haploid embryo selection during doubled haploid breeding of maize.

**Supplementary Information:**

The online version contains supplementary material available at 10.1186/s12870-022-03971-5.

## Background

Doubled haploid (DH) technology has dramatically improved the breeding efficiency and shortened the breeding process in maize [1]. Identification of haploids is a crucial step in DH breeding. Several methods of haploid selection at the post-emergence stage have been reported; red root [2, 3], purple sheath and plant color [4, 5] are employed as markers for the validation of putative haploids. The identification of haploids at the seed stage could substantially reduce the number of plants being handled at later stages in the process of DH production [6].

The difference between haploid and diploid seeds lies mainly in the genetic constitution of their embryos. With the application of immature embryo-based chromosome doubling in breeding programs, separation of immature haploid embryos from diploid embryos is increasingly important. To date, haploid inducers such as RWS-GFP have been developed that contain fluorescent proteins expressed specifically in embryos [7]. Nevertheless, special equipment is needed to screen fluorescent embryos, complicating the selection procedure. In addition, molecular markers [8] and flow cytometry [9] have been employed to verify the ploidy level but these methods are not appropriate for large-scale haploid identification in breeding programs.

Compared with fluorescent markers, the identification of haploids based on a visual anthocyanin marker is more convenient [10]. Two classes of genes, structural and regulatory genes, are involved in the anthocyanin biosynthesis pathway. The transcription of structural genes is regulated by transcription factors, including *colorless 1* (*C1*), *purple leaf 1* (*PL1*), *booster 1* (*B1*), *red color 1* (*R1*) and *pale aleurone color 1* (*PAC1*), which form the MYB transcription factor/basic helix-loop-helix (bHLH) domain protein/WD-repeat (MYB-bHLH-WD) transcription factor complex [11]. Among these genes, *R1-navajo* (*R1-nj*, an allele of *R1*) results in pigmentation of the scutellum and aleurone, and has been used for the selection of mature haploid seeds [6]. Structural genes involved in anthocyanin biosynthesis, including *anthocyaninless 1* (*A1*), *anthocyaninless 2* (*A2*), *colorless 2* (*C2*), *bronze 1* (*Bz1*), and *bronze 2* (*Bz2*), are essential for the expression of *R1-nj*, for which the regulatory gene *C1* is also required [12]. Anthocyanin expression can vary significantly among germplasm sources after crossing with a haploid inducer [13]. Some tropical and subtropical maize germplasms carry inhibitory alleles of genes involved in the anthocyanin pathway, such as *C1-I* [14], *C2-Idf* [15] and *In1-D* [16], and are thus disadvantageous for haploid screening. In addition, the accumulation of anthocyanin is affected by other factors, including haploid inducers, developmental stage and the growing environment [13, 17, 18]. Immature haploid embryos have been widely used in DH production due to their high efficiency of chromosome doubling [19]. Hence, the identification of haploids at the immature embryo stage is critically important for chromosome doubling in immature embryos. To date, few researchers have attempted to identify the influencing factors and mechanism of immature haploid embryo selection based on anthocyanin markers.

In this study, we compared factors related to anthocyanin synthesis that affect the pigmentation of embryos, and demonstrated that light is an essential factor in the identification of immature haploid embryos. Meanwhile, transcriptome analyses revealed differentially expressed genes (DEGs) involved in anthocyanin biosynthesis, which control anthocyanin accumulation in immature embryos, under light stimulation. The results of this study provide a useful guide for further improving the efficiency of haploid identification, thereby facilitating the development of DH technology.

## Results

### Factors influencing anthocyanin accumulation in immature diploid embryos

**Fig. 1:**
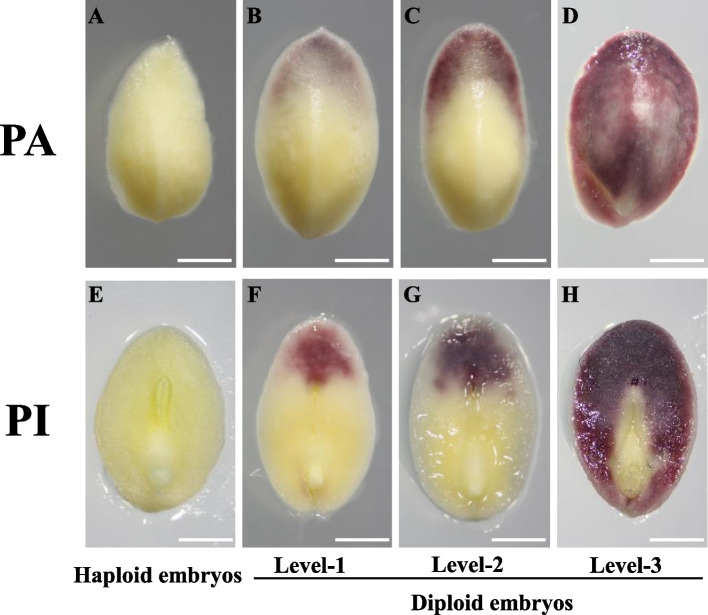
Different levels of PA (**B**–**D**) and PI (**F**–**H**) in diploid embryos derived from ZD958×CAU5. **A** and **E** show immature haploid control embryos. **B**–**D** PA grading according to the percentage of pigmented area on the scutellum. **B** level-1, 0–25%; **C** level-2, 25–50%; **D** level-3, 50–100%. **F**–**H** PI grading according to the degree of pigmentation of the scutellum. **F** level-1, pink; **G **level-2, red; **H** level-3, purple. Scale bars, 1 mm (**A**–**H**)

**Fig. 2:**
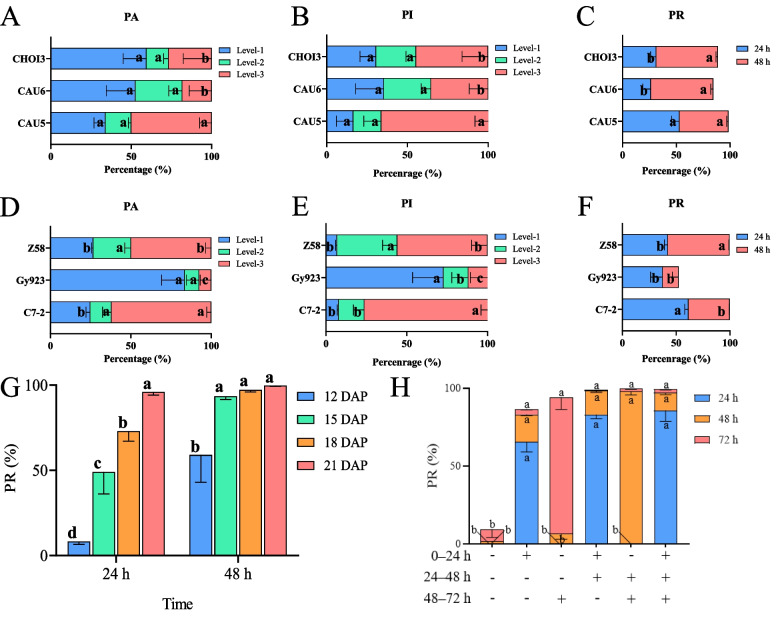
Evaluation of factors influencing induction cross embryo pigmentation. **A**–**C** Effect of paternal genetic background on embryo coloration in ZD958. **D**–**F** Effect of maternal genetic background on the pigmentation of embryos derived from CAU5 induction. **G** Effect of embryo age on the pigmentation of embryos derived from B73×CAU5. **H** Effect of light on the pigmentation of embryos derived from B73×CAU5. The symbols + and - represent the light and dark treatments, respectively. Different lowercase letters indicate a significant difference among groups at *p* < 0.05 (Tukey’s honestly significant difference [HSD]).

Immature embryos derived from ZD958×CAU5 with various pigmentation area (PA) and pigmentation intensity (PI) values are shown in Fig. 1. To study the influences of various inducers, the female parent, embryo age and light exposure on the pigmentation of embryos, we used three haploid inducer lines, CHOI3, CAU6 and CAU5 to pollinate the commercial hybrid ZD958. As shown in Fig. 2A and B, among the colored embryos derived from ZD958×CAU5, embryos at level–3 of PA and PI accounted for 50.0% and 66.2% of all embryos, respectively, and these proportions were significantly higher than the values for embryos derived from ZD958×CAU6 and ZD958×CHOI3. In addition, embryos from ZD958×CAU5 had a significantly higher 24 h pigmentation rate (PR) than embryos from the other two induction crosses (Fig. 2C). Next, we analyzed the effect of different female parents on pigmentation. The results revealed that, among the three inbred lines pollinated by CAU5, the inbred lines Z58 and C7-2 had much stronger pigmentation phenotypes than GY923 (Fig. 2D, F). In contrast to C7-2 and Z58, which had relatively low proportions of level-1 PA and PI embryos, the proportions of level-1 PA and level-1 PI embryos reached 83.5% and 72.7%, respectively, in Gy923. However, the proportions of level-3 PA embryos and level-3 PI embryos were significantly higher in Z58 and C7-2 than in GY923. This result suggests that Gy923 may exist inhibitory factors that hinder anthocyanin accumulation. Finally, GY923 had a significantly lower 48 h PR than Z58 and C7-2. These results indicate that both male and female parents influence the pigmentation of embryos, and that CAU5 is superior in terms of embryo anthocyanin accumulation.

To observe the influence of embryo age on anthocyanin accumulation, diploid embryos derived from B73×CAU5 were isolated at 12, 15, 18 and 21 days after pollination (DAP), respectively. At 24 h after light exposure, PR was strongly correlated with embryo age, as the PR of embryos at 12 DAP was only 8.3%, while that of embryos at 21 DAP reached 96.0% (Fig. 2G). At 48 h after light exposure, the average PR of embryos at 12 DAP was 59.1%, which was significantly lower than the corresponding value for embryos at 15–21 DAP. No difference was found among the PR values of embryos at 15, 18 and 21 DAP after 48 h of light exposure. In summary, the optimal age for haploid embryo identification is at least 15 DAP.

Immature embryos derived from B73×CAU5 at 15 DAP were used to evaluate the influence of light on anthocyanin accumulation. The results revealed that the PR in the three groups without light exposure for 0–24 h was in the range of 0.0–0.3%, which was significantly lower than the PR of 72.0–85.7% with light treatment for the second, fourth and sixth groups (Fig. 2H). When embryos were shifted from initial dark to light conditions at 24–48 h, the 48 h PR improved, with a value of 97.9% in the fifth treatment; a similar result was obtained in the third treatment, which was cultured in the dark for 48 h and then shifted to light conditions for 24 h. In contrast, the first and third treatments were continuously cultured in the dark for 48 h, and their PR values were below 10.0%. After being cultured in the light for 48 h, the PR of the fourth and sixth treatments increased to above 97.0%, indicating that the pigmentation of most diploid embryos was complete. Interestingly, the PR continued to increase in the second treatment, even after it was transferred from light to dark, and the same pattern was observed in the fourth treatment at 48–72 h. The 72 h PR was only 9.4% for the sixth treatment, which was cultured fully in the dark, whereas the first treatment with continuous lighting had a 72 h PR of 99.4%. These results demonstrate that light can activate anthocyanin accumulation in diploid embryos and plays a decisive role among the factors investigated.

### Identification accuracy (IA) of immature haploid embryos under various conditions

**Fig. 3:**
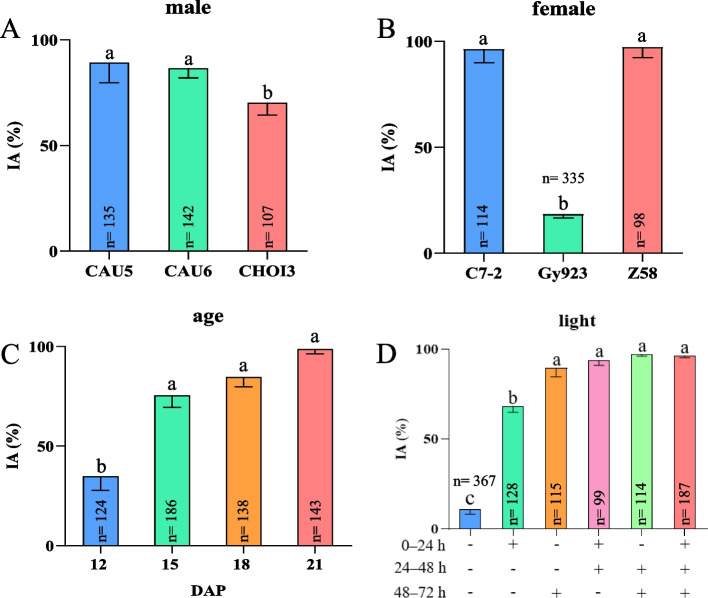
Accuracy of immature haploid embryo identification with various influencing factors after culture in vitro. The putative immature haploid embryos in **A**–**C** were selected after culturing for 48 h. The putative immature haploid embryos in **D** were selected after culturing for 72 h. The symbols + and - represent the light and dark treatments, respectively. Different lowercase letters indicate a significant difference among groups at *p* < 0.05 (Tukey’s HSD)

The IA of immature haploid embryos depended on anthocyanin accumulation in the diploid embryos. We analyzed the IA of immature haploid embryos under various conditions. The results showed that the haploid inducer line has a strong impact on the IA of immature haploid embryos. The average IA values of CHOI3, CAU6 and CAU5 were 70.2%, 86.5% and 89.2%, respectively. The IA of CHOI3 was significantly lower than the values of CAU5 and CAU6, while no difference was apparent between CAU5 and CAU6 (Fig. 3A). On the other hand, the IA of immature haploid embryos correlated with the pigmentation performance of the female genetic background, as the IA values of C7-2 and Z58 were 96.3% and 97.2%, respectively, which were markedly higher than the IA of 18.4% for Gy923 (Fig. 3B). In terms of embryo age, the IA improved from 34.8% to 12 DAP to 75.5% at 15 DAP, and then rose above 80.0% at 18 and 21 DAP (Fig. 3C). Finally, exposure to light substantially improved the IA, as the statistical results (Fig. 3D) showed that the IA in the dark was 11.0%, which improved to 96.4% after 72 h of exposure to light. More importantly, the IA corresponded to the timing of light exposure. The IA was above 90% after 48 h of exposure to light, but was only 68.4–89.7% after 24 h of exposure to light.

### Expression profiling and pathway annotation in immature diploid embryos exposed to light

**Fig. 4:**
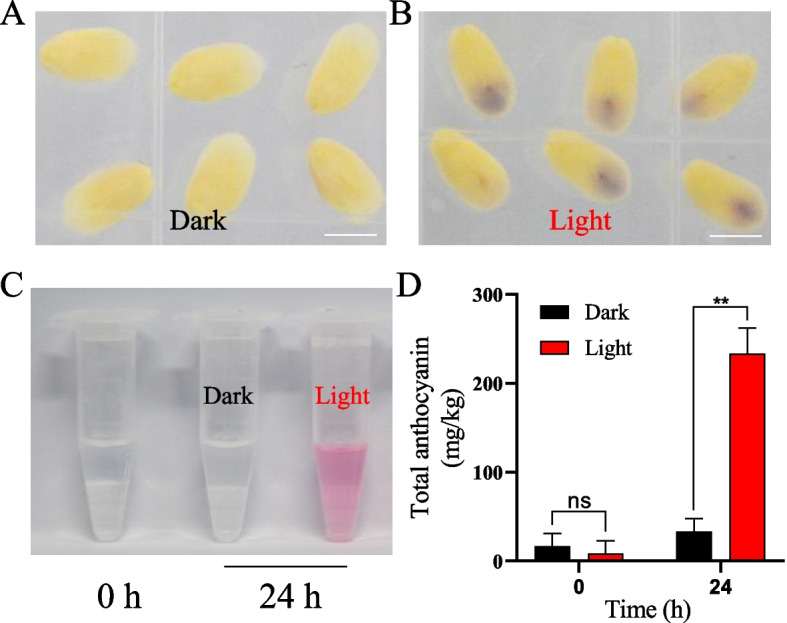
Anthocyanin accumulation and quantification in diploid embryos at 15 DAP derived from B73×CAU5 after culturing for 24 h under dark and light conditions. **A**–**B** Coloration of diploid embryos after culturing for 24 h under dark or light conditions. **C** Anthocyanin accumulation in diploid embryos grown in dark and light environments. **D** Anthocyanin quantification in diploid embryos grown under dark and light conditions at 0 and 24 h. Scale bars, 2 mm (**A**–**B**). ** represents *p* < 0.01 in the comparison of total anthocyanin content between dark and light conditions (two-tailed *t* test)

The results described above indicate that light exposure is one of the most important factors affecting immature embryo pigmentation. We next investigated the genes and pathways involved in light-induced pigment accumulation. To this end, we isolated embryos at 15 DAP from B73×CAU5, which were cultured for 24 h under light and dark conditions. Apparent pigment accumulation was observed in the embryos cultured with light exposure, but not in the embryos cultured in a dark environment (Fig. 4A, B). We quantified the total anthocyanin content of each embryo from both groups, and the results showed no difference between the light and dark treatments before culturing (Fig. 4D). However, anthocyanin accumulation improved significantly to 233.3 mg/kg after 24 h of light exposure, which is nearly 8-fold higher than the anthocyanin content of embryos grown in the dark (Fig. 4C, D).

**Fig. 5:**
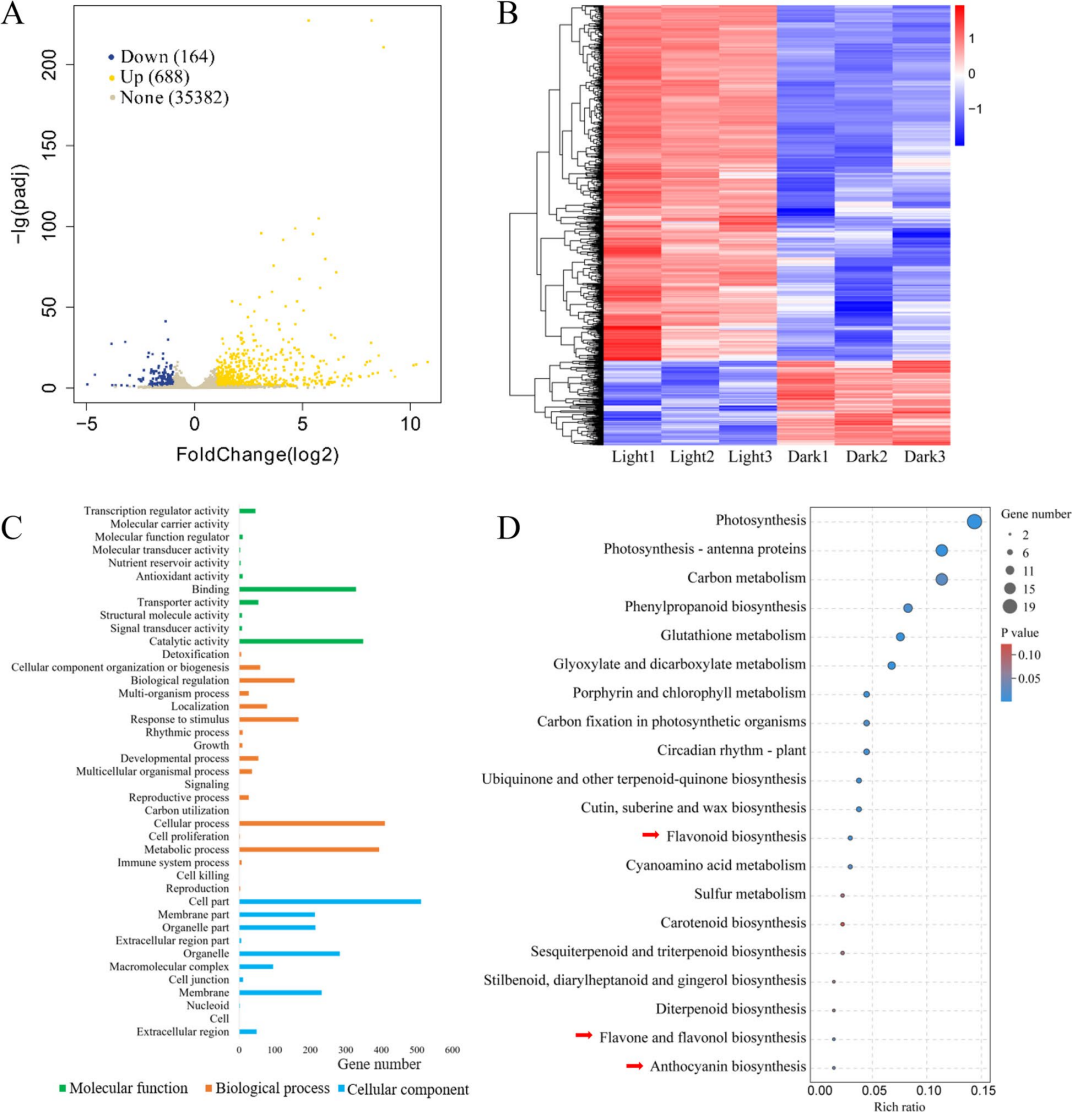
Gene expression profiles of diploid embryos derived from B73×CAU5 in response to dark and light conditions. **A** Diversity analysis of up- and down-regulated genes shown as a volcano plot. The data for all genes are plotted as log2 fold changes versus the −log10 adjusted *p*-value. Blue, yellow and gray points represent down-regulated genes, up-regulated genes and genes with expression levels that did not significantly differ between dark and light environments. **B** Clusters of DEGs between dark and light conditions. Genes are shown horizontally; each column represents a sample. Genes with high and low expression are shown in red and blue, respectively. **C**Significantly enriched GO terms (*p *< 0.05) in the DEGs. **D** Top 20 KEGG pathways enriched in the annotated DEGs. Rich ratio represents the ratio of differential expression for the top 20 DEGs

Next, a comparative transcriptome analysis of the immature embryos was performed. As shown in the volcano plot, the fragments per kilobase million (FPKM) value for 32,608 total genes were obtained through transcriptome sequencing, of which 852 exhibited significant expression level differences between the dark and light treatments based on the criteria of |(log2(fold change)|> 1 and *P*-adjust value < 0.05. Among 852 DEGs, 688 were up-regulated and 164 were down-regulated by light exposure (Supplementary Table S1 and Fig. 5A). The heat map of these DEGs indicated good repeatability between biological replicates (Fig. 5B).

To identify the putative pathways associated with these DEGs, a Gene Ontology (GO) enrichment analysis was performed and 41 functional terms were identified. The DEGs were enriched mainly in terms associated with cellular components such as cell part, organelle and membrane part. For the biological process category, the genes were mostly enriched in the metabolic process, cellular process, biological regulation and response to stimulus terms. Within the molecular function category, the DEGs were commonly enriched in the terms catalytic activity, binding and transcription regulator activity (Fig. 5C and Supplementary Table S2). Previous studies have shown that light affects anthocyanin biosynthesis via the processes of light signal transduction and transcriptional regulation [20]. Therefore, we focused on functional subgroups related to anthocyanin biosynthesis, including the terms metabolic process, signaling, transcription regulator activity and transporter activity, from among the 41 functional terms identified as significant.

To further explore the biological functions of the DEGs, an enrichment analysis was performed using the Kyoto Encyclopedia of Genes and Genomes (KEGG) database (Fig. 5D and Supplementary Table S3). Compared to dark treatment, 119 significant DEGs were identified in diploid embryos exposed to light. Among these 119 DEGs, 100 were up-regulated and 19 were down-regulated. With continuous (24 h) light exposure, most significant DEGs were distributed in the photosynthesis and plant circadian rhythm pathways. More importantly, six genes related to the anthocyanin biosynthesis pathway were identified, including five up-regulated genes-namely, chalcone synthase (*CHS*), anthocyanidin synthase (*ANS*), flavanone 3′-hydroxylase (*F3′H*), anthocyanidin 3-O-glucosyltransferase (*3GT*), and flavonoid 3′-monooxygenase (*Zm00001d007918*), as well as one down-regulated gene, malonyl-CoA (*Zm00001d052042*).

### Selected anthocyanin biosynthesis-related genes in immature diploid embryos

**Fig. 6:**
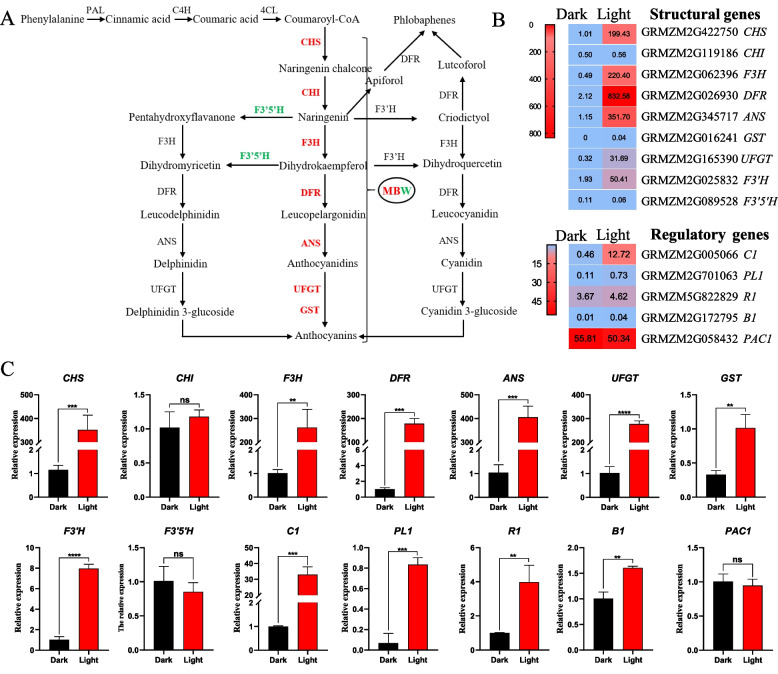
DEGs related to anthocyanin biosynthesis in immature diploid embryos derived from B73×CAU5 under dark and light conditions. **A** Simplified map of the anthocyanin biosynthesis pathway. Red indicates up-regulated genes and green indicates down-regulated genes. MBW represents the MYB-bHLH-WD transcription factor complex. **B** Expression profile of genes related to anthocyanin biosynthesis in immature diploid embryos. The color indicates the FPKM value of the genes (high: red, low: blue). **C** Relative expression levels of 14 genes associated with the anthocyanin biosynthetic pathway obtained through qRT-PCR. Data represent the mean ± standard deviation; ns, not significant, ** represents *p*-value < 0.01 and *** represents *p*-value < 0.001 for the comparison of values between dark and light conditions (two-tailed *t* test)

The expression levels of 14 genes related to the anthocyanin biosynthesis pathway, including 9 structural genes and 5 regulatory genes, were compared between the dark and light groups using RNA sequencing (RNA-seq) data (Fig. 6 and Supplementary Table S4). As shown in Fig. 6A and B, the expression levels of the structural gene flavonoid 3′,5′-hydroxylase (*F3′5′H*) and the regulatory gene *PAC1* were down-regulated. All other investigated genes, including the structural genes *CHS*, flavanone 3-hydroxylase (*F3H*), dihydroflavonol reductase (*DFR*), *ANS*, uridine diphosphate glucose flavonoid 3-glucosyltransferase (*UFGT*), glutathione S-transferase (*GST*) and *F3′H*, as well as the regulatory transcription factors *C1*/*PL1* and *R1*/*B1*, were up-regulated by light exposure. Furthermore, *DFR* and *ANS*, two structural genes involved in the anthocyanin biosynthesis pathway, showed markedly increased transcript levels. In addition, a high expression level of *F3H* may contribute to the accumulation of anthocyanins. As noted above, the expression levels of structural genes are controlled by the MYB-bHLH-WD transcription factor complex. The expression of *C1* was significantly up-regulated, whereas the up-regulation of *PL1*, *R1* and *B1* was slight and not significant (based on FPKM) and was significant based on qRT-PCR. In the terms of differential expression level, *C1* was the key genes regulating anthocyanin biosynthesis in immature embryos.

To verify the expression levels of these 14 genes involved in anthocyanin biosynthesis, quantitative reverse transcription polymerase chain reaction (qRT-PCR) was performed (Fig. 6C) and the primers used were listed in Supplementary Table S5. The results showed that the expression patterns were consistent between qRT-PCR and RNA-seq.

## Discussion

### Factors influencing the identification of immature haploid embryos

Anthocyanin has long been employed in the identification of haploids. The performance of anthocyanin markers, mainly *R1-nj*, in mature haploid seeds has been studied extensively, and is significantly affected by the genetic background of the inducer (male parent) and the source germplasm (female parent) [10, 13, 17]. The identification of mature haploid seeds can be influenced by haploid inducer lines. Different degrees of pigmentation were present on the endosperm and embryo of diploid seeds after a given source germplasm was crossed with different *R1-nj*-based haploid inducers [21]. Poor pigmentation of the endosperm and embryo would seriously affect the classification accuracy of diploids, haploids and pollen-polluted seeds. For example, the identification of mature haploid seeds is not feasible in black or purple waxy corn due to its colored pericarp, which impedes haploid selection.

Nevertheless, with the development of immature embryo-based chromosome doubling methods, the chromosome doubling rate was improved significantly [19], the identification of immature haploid embryos has become crucial. In this study, we found that different female parents produced differing pigmentation phenotypes in immature embryos, consistent with previous research on the pigmentation of mature seeds [13]. However, we found that embryo age significantly affected the pigmentation of diploid embryos, with the optimal age for haploid embryo identification being after 15 DAP, and that the anthocyanin marker is stable at the mature seed stage. In addition, embryo pigmentation was strongly affected by the culture environment; among the factors we tested, light played crucial role in the pigmentation of diploid embryos and IA of immature haploid embryos. To date, few studies have reported the effect of light on the pigmentation of seeds during the identification of haploids using the *R1-nj* marker system. Light-induced anthocyanin accumulation has been observed not only in maize but also in pear [22, 23], *Arabidopsis* [24] and tomato [25]. Aside from the factors tested in the present study, temperature [26], gibberellins, abscisic acid [27] and sucrose [28] improve anthocyanin accumulation, and thus are potential factors for further enhancing embryo coloration [29].

### Regulatory mechanism of anthocyanin synthesis during haploid identification

The regulatory mechanism underlying anthocyanin synthesis has been well studied in maize, but the pigmentation of diploids during haploid identification remains unclear. High expression levels of structural genes, including *CHS*, *F3H*, *DFR* and *ANS*, are important for anthocyanin accumulation, and the expression levels of these structural genes are controlled by transcription factors [30]. The expression of *CHS* and *DFR* is promoted by blue light, as reported in the ray florets of *Gerbera hybrida* [31]. Similarly, blue light enhanced the activities of *CHS*, *F3H*, *DFR*, *ANS* and *UFGT* in postharvest strawberries [32]. High expression of *F3′H* combined with low expression of *F3′5′H* led to the accumulation of cyanidin 3-glucoside anthocyanin, and *F3′H* is activated by *C1* and *R1* in the aleurone of maize seeds [33]. Our results reveal that among the transcription factors involved in anthocyanin biosynthesis, *C1* was significantly up-regulated after exposure to light, signifying that *C1* might be a key gene in enhancing the expression of structural genes in the anthocyanin biosynthesis pathway, as reported previously [20, 34]. *R1* is another critical gene in anthocyanin synthesis [34]. In our study, the expression of *R1* was not altered significantly by light exposure (based on FPKM), as *R* gene expression cannot be induced by light, in accordance with previous results [34].

Aside from environment factors, anthocyanin synthesis can be improved through regulation of the expression of two types of transcription factors. *C1* in seeds and *PL1* in plant tissues contribute to the light-dependent regulation of anthocyanin accumulation, while bHLH genes such as *R1*, *B1*, *scutellar node1* (*Sn1*), *leaf color 1* (*Lc1*) and *Hopi1* determine the tissue-specific synthesis of anthocyanins [35]. The MYB *C1*/*PL1* and bHLH *R1*/*B1* gene families have been used to improve the anthocyanin contents of seeds and other tissues; for example, the pericarp of rice became dark brown when transformed with a construct of *ZmC1* and *ZmR-S* driven by a promoter from the rice prolamin-encoding gene [36], and overexpression of the transcription factors *ZmC1* and *ZmR* in wheat led to the development of anthocyanin-enriched wheat germplasms [37]. Further, black Mexican sweet maize cells expressed the regulators *R* and *C1* under the *CaMV 35 S* constitutive promoter, leading to accumulation of anthocyanins with no requirement for additional light-induced regulators [38]. Furthermore, purple embryo maize was created through the establishment of a multigene expression system including *C1*, *R2*, *Bz1* and *Bz2* [39].

### Optimization of haploid identification and improvement of DH technology efficiency

To improve the efficiency of haploid identification using anthocyanins as a marker, the expression of genes involved in anthocyanin synthesis must be enhanced through technological and biological means. Light can activate the expression of genes involved in anthocyanin biosynthesis [39, 40]. In terms of biological means of improving anthocyanin production, the development of a new haploid inducer with an enhanced anthocyanin marker designed for immature haploid embryo identification was an effective method to improve the efficiency of immature haploid embryo identification [21]. To eliminate the negative effects of color inhibition gene effects on pigmentation in the aleurone layer and embryo of some germplasms, haploid inducers known as Maize Anthocyanin Gene InduCers (MAGIC) were developed through the co-expression of *ZmC1* and *ZmR2* in our previous study [41]. Overall, the present findings of factors influencing pigmentation in immature embryos will improve the efficiency of haploid identification in DH technology.

## Conclusion

In this study, we systematically analyzed the effects of factors including the haploid inducer line, female parent, embryo age and light exposure on the pigmentation of immature embryos. Our results demonstrate that light exposure is crucial to promoting embryo pigmentation. Transcriptome analysis revealed that DEGs are enriched in the flavonoid, flavone and flavonol and anthocyanin biosynthesis pathways. The expression levels of *F3H*, *DFR*, *ANS*, *F3′H* and the transcription factor *C1*, which are involved in anthocyanin biosynthesis, were significantly up-regulated, suggesting roles for these genes in anthocyanin accumulation. The findings of this study provide new insight into anthocyanin accumulation in immature embryos, and will be valuable for further improving haploid embryo identification efficiency.

## Materials and methods

### Plant materials

Three haploid inducers, CAU5, CAU6 and CHOI3, were used as the male parents in this study. We used four inbred lines, B73, Chang7-2 (C7-2), Gaoyou923 (Gy923) and Zheng58 (Z58), along with the hybrid Zhengdan958 (ZD958), to evaluate embryonic anthocyanin accumulation and the efficiency of haploid embryo identification. Our previous results showed that the tester lines C7-2, Gy923 and Z58, showed different degree of coloration and thus result in the difference of accuracy in identification of haploid seeds. All materials were grown at the experimental station of Beijing Academy of Agriculture and Forestry Sciences and China Agricultural University. Unless stated, all embryos were isolated at 15 DAP.

### Embryo isolation and culture

Embryo isolation and in vitro culture were performed according to published protocols [42]. The ears derived from maternal materials crossed with haploid inducers were harvested at 12–21 DAP. 75% ethyl alcohol was used to sterilize the ears. Immature embryos were isolated from the sterile seeds manually. Thereafter, embryos were immediately cultured in Murashige and Skoog medium (containing 30 g/L of sucrose, 7.5 g/L of agar and 2% dimethyl sulfoxide [pH 5.8]) with the embryo axis facing upward. Then, all embryos were cultured in a growth chamber at 28ºC and 60% relative humidity. After 24–72 h, anthocyanin accumulation was evaluated according to the area and intensity of pigmentation in the embryos. In this process, haploid embryos exhibited no pigmentation due to the absence of the anthocyanin marker.

We analyzed the effects of four factors (the male parent, female parent, embryo age and light exposure) on embryo pigmentation. To analyze the effect of the male parent on immature embryo pigmentation, we pollinated three haploid inducers, CAU5, CAU6 and CHOI3, to ZD958; the resulting embryos were isolated and cultured for 24 h under light. We used CAU5 as the male parent for crossing with three inbred lines, C7-2, Gy923 and Z58, to analyze the effect of the female parent. To study the effect of embryo age on embryo pigmentation, we isolated immature embryos from the B73×CAU5 cross at 12, 15, 18 and 21 DAP, and cultured them for 24 or 48 h under light conditions. In the light exposure experiment, we set three time widows, 0–24 h, 24–48 h and 48–72 h, with independent light exposure (+) and dark control (-) groups for each time window, constituting six treatments: 0–72 h, dark (---); 0–24 h, light and 24–72 h dark (+--); 0–48 h, dark and 48–72 h, light (--+); 0–48 h, light and 48–72 h, dark (++-); 0–24 h, dark and 24–72 h, light (-++); and 0–72 h, light (+++).We chose the incandescent lighting,, with quantity of 20,000 lx. The PR and PI of all embryos were evaluated visually, and the PR was calculated using the formula PR (%) = (number of pigmented embryos/total number of embryos) × 100%. Pigmentation intensity (PI) were employed based on visual scoring of the percentage of the scutellum area that was pigmented and the degree of pigmentation of the scutellum, respectively. The immature embryos were classified into PA levels 1–3 based on the thresholds of PA < 25%, 25% < PA < 50% and PA > 50%, respectively. For PI, levels 1–3 exhibited pink, red and purple pigmentation, respectively. In addition, putative haploid embryos identified were verified based on plant characters in the field; the identification accuracy was calculated to evaluate the efficiency of haploid identification based on immature embryo pigmentation.

### Embryo anthocyanin quantification

Immature embryos were ground into a fine powder under liquid nitrogen and dried at − 80 °C. To observe the total anthocyanin content of immature B73×CAU5 embryos under various culture conditions, a spectrophotometric method was used as described previously with cyanidin 3-O-glucoside (CAS: 7084-24-4) as the standard. An extraction solution was prepared by mixing 1% HCl with 90% ethanol at a volume ratio of 1:1. The operating solution was prepared at a sample-to-extraction solution volume ratio of 1:15 and subjected to ultrasonic extraction for 1 h, and then the supernatant was collected following centrifugation at 12,000 rpm for 10 min. The absorbance of a mixture of 1.0 mL of supernatant with 4.0 mL of sodium acetate buffer (pH 4.5) was measured on an ultraviolet-visible spectrophotometer at wavelengths of 520 and 700 nm. The anthocyanin concentration (mg/L) was calculated as follows: C = Af /(εL)×103×MW, where A is the absorbance difference between two pH conditions, f is the dilution factor, L is the light path of the cuvette (cm), ε is the molar absorptivity (26,900 M^− 1^ cm^− 1^) and MW is the molar mass (484.84) of the selected standard [43, 44].

### Sample collection and preparation for RNA-seq

Hybrid embryo samples of the B73×CAU5 cross were isolated at 15 DAP, which is a common analysis time for immature embryo identification [21], and cultured at 28 °C and 60% relative humidity. The embryos in the dark control were completely protected from light by a covering of silver paper. Embryos in the light treatment were cultured under a light intensity of 20,000 lx for 24 h. Three independent biological replicates were sampled immediately and frozen in liquid nitrogen and until RNA extraction.

### RNA-seq analysis

Total RNA extraction and quality control were performed according to standard protocols. Annoroad Gene Technology Co. (Beijing, China) constructed the cDNA library and performed Illumina sequencing on a HiSeq 4000 instrument (Illumina, San Diego, CA) to yield 150-bp paired-end reads. The number of clean reads mapped to a specific gene were counted and then presented as fragments per kilobase of transcript per million mapped reads (FPKM) using Cufflinks [45]. Differentially expressed genes (DEGs) between the light treatment and the dark treatment samples were identified using the DESeq software [46]. Significant DEGs were identified through a comparison of FPKM values between the dark and light groups with the criteria *p* < 0.01 and |Log2FoldChange| ≥ 1. Gene function was annotated according to GO and KEGG databases (http://www.genome.jp/kegg/) [47]. GO enrichment and KEGG enrichment of DEGs was determined based on the Wallenius noncentral hypergeometric distribution using the GOseq R package [48] and KOBAS software [49], respectively. The significance of GO and KEGG term enrichment was estimated using the hypergeometric test (*p* < 0.05).

### qRT-PCR

Total RNA was manually isolated with TRIzol reagent (DP441; Tiangen, Beijing, China). cDNA was synthesized from 1 µg of RNA using a PrimeScript™ RT Kit with gDNA Eraser (RR047A; Takara Bio Inc., Otsu, Japan). qRT-PCR was performed using SYBR Premix ExTaq (RR820A; Takara Bio Inc.). The PCR cycling conditions were: 95 °C for 30 s, followed by 39 cycles of 95 °C for 10 s and 60 °C for 30 s, followed by 95 °C for 10 s, followed by melt curve 65 to 95 °C increment o.5 °C for 5 s. Expression levels were calculated relative to the reference gene (ubiquitin protein ligase) using the formula 2^(−ΔCT)^. Three biological and three technical replicates were performed for all RT-qPCR experiments.

## Supplementary Information

**Additional file 1: Supplementary Table S1.** Annotation of DEGs identified in light vs dark.

**Additional file 2: Supplementary Table S2.** GO pathway analysis of DEGs under light.

**Additional file 3: Supplementary Table S3.** KEGG pathway analysis of DEGs under light.

**Additional file 4: Supplementary Table S4.** List of anthocyanin biosynthesis-related maize genes.

**Additional file 5: Supplementary Table S5.** Primers used for RT-qPCR in this study.

## Data Availability

The original contributions presented in the study are publicly available. The RNA-seq data have been deposited in the NCBI Sequence Read Archive, accession number: PRJNA868492 (https://www.ncbi.nlm.nih.gov/sra/PRJNA868492).

## Abbreviations

DH: Doubled haploid
MYB-bHLH-WD: MYB transcription factor/basic helix-loop-helix (bHLH) domain protein/WD-repeat
DEGs: Differentially expressed genes
PA: Pigmentation area
PI: Pigmentation intensity
PR: Pigmentation rate
DAP: Days after pollination
IA: Identification accuracy
FPKM: Fragments per kilobase million
GO: Gene Ontology
KEGG: Kyoto Encyclopedia of Genes and Genomes
