# Supplementary material for: Influential factors and transcriptome analyses of immature diploid embryo anthocyanin accumulation in maize
Source: BMC Plant Biol. 2022 Dec 24;22:609. doi: 10.1186/s12870-022-03971-5 (PMC9789580; doi:10.1186/s12870-022-03971-5)
Supplement: Supplementary file 4 — Additional file 4: Supplementary Table S4. List of anthocyanin biosynthesis-related maize genes. [file 12870_2022_3971_MOESM4_ESM.docx]

**Supplementary Table S4. List of anthocyanin biosynthesis-related maize genes**

| **Function classification** | **Protein** | **Gene name** | **Gene symbols** | **Synonyms** |
| --- | --- | --- | --- | --- |
| **Structure genes** | Dihydroflavonol reductase(DFR) | *Anthocyaninless 1* | *A1* | GRMZM2G026930 |
|  | Anthocyanidin synthase(ANS) | *Anthocyaninless 2* | *A2* | GRMZM2G345717 |
|  | UDP-glucose flavonoid 3-glucosyltransferase(UFGT) | *Bronze 1* | *Bz1* | GRMZM2G165390 |
|  | Glutathione S-transferase(GST) | *Bronze 2* | *Bz2* | GRMZM2G016241 |
|  | Chalcone synthase(CHS) | *Colorless 2* | *C2* | GRMZM2G422750 |
|  | Chalone isomerase (CHI) |  | *CHI* | GRMZM2G119186 |
|  | Flavanone 3-hydroxylase(F3H) | *Fht1* | *F3H* | GRMZM2G062396 |
|  | Flavonoid 3'5'-hydroxylase (F3'5'H) |  | *F3'5'H* | GRMZM2G089528 |
|  | Flavonoid 3'-hydroxylase (F3'H) | *Red aleurone 1* | *Pr1* | GRMZM2G025832 |
|  |  |  |  |  |
| **Regulatory genes** | MYB transcriptional factor | *Colorless1* | *C1* | GRMZM2G005066 |
|  |  | *Purple Plant 1* | *PL1* | GRMZM2G701063 |
|  | bHLH transcriptional factor | *Red color 1* | *R1* | GRMZM5G822829 |
|  |  | *Booster 1* | *B1* | GRMZM2G172795 |
|  | WD40 | *Pale Aleurone Color 1* | *PAC1* | GRMZM2G058432 |
