# Supplementary material for: Influential factors and transcriptome analyses of immature diploid embryo anthocyanin accumulation in maize
Source: BMC Plant Biol. 2022 Dec 24;22:609. doi: 10.1186/s12870-022-03971-5 (PMC9789580; doi:10.1186/s12870-022-03971-5)
Supplement: Supplementary file 5 — Additional file 5: Supplementary Table S5. Primers used for RT-qPCR in this study. [file 12870_2022_3971_MOESM5_ESM.docx]

**Supplementary Table S5. Primers used for RT-qPCR in this study**

| **Primer** | **Sequence** |
| --- | --- |
| A1F | 5'-TGTCCAAAGACCCTGAGAATGAGG-3' |
| A1R | 5'-GCATGCCCGCATGATGCTTATC-3' |
| A2F | 5'-ATTCAAGCAGCACCTGGATCGC-3' |
| A2R | 5'-AGACATGTGCAGCCCATCAGTTG-3' |
| B1F | 5'-GCTTCATGAGGCTCAAGATAGG-3' |
| B1R | 5'-GCCCTTTCATCGCTTCCCTA-3' |
| Bz1F | 5'-GGTGAGTGCAGGAAGAACTTCGAC-3' |
| Bz1R | 5'-TGAACAGCAAGACGACCTTTCACG-3' |
| Bz2F | 5'-ACCGGCTCCCGTTTAAATTGCC-3' |
| Bz2R | 5'-TCGACGTGACTTCTGTTGGCATC-3' |
| C1F | 5'-ACTCTTCTTCTTCCACCGGGACAC-3' |
| C1R | 5'-AATCTTCCGACGACCCTGCTTC-3' |
| C2F | 5'-TCATGCAAGACCAGGGTAGAGC-3' |
| C2R | 5'-CCCATGCATGCTAAAGTGACAACG-3' |
| CHIF | 5'-AGGAGAAAGAGAACCGATCAAG-3' |
| CHIR | 5'TTCAAATGTTACCACAGCGAAC-3' |
| F3HF | 5'-CGACAAGAGTGCCAACAAGGAG-3' |
| F3HR | 5'-ATCAGGCAAGAATGGCGTCGAG-3' |
| Pr1F | 5'-ATGAACGGGATCATCAGGGAGAGG-3' |
| Pr1R | 5'-TCAGGAGAAGTGCCTTGATGTCAG-3' |
| F3'5'HF | 5'-ACATGCTCAAGCTCGAGAAGGAAG-3' |
| F3'5'HR | 5'-CGACCACCATGTCCATTAGCAG-3' |
| PL1F | 5'-CTACGACGAGGAGGATCTCATC-3' |
| PL1R | 5'-TGCTGTTCCAGTAGTTCTTGAT-3' |
| PAC1F | 5'-AACCCGGACGTTGTTATGGAACAG-3' |
| PAC1R | 5'-AAAGCACTTGTTCCCGACGGTCTC-3' |
| R1F | 5'-AGCTCAACGAGATGTTCCTCGTC-3' |
| R1R | 5'-TCGACGCTTTGTTCACCCTGTG-3' |
| Zm-actinF | 5'-GGGATTGCCGATCGTATGAG-3' |
| Zm-actinR | 5'-GAGCCACCGATCCAGACACT-3' |
